# Supplementary material for: Acute Effect of Three Aerobic Exercise Intensities on Glomerular Filtration Rate in Healthy Older Adults
Source: Diseases. 2024 Oct 11;12(10):249. doi: 10.3390/diseases12100249 (PMC11507922; doi:10.3390/diseases12100249)
Supplement: Supplementary file 1 [file diseases-12-00249-s001.zip › diseases-3230117-supplementary.pdf]

Supplementary File

Table S1. Equations to estimate filtration glomerular rate by serum creatinine and cystatin C.

Gender		Serum Creatinine (SCr), $mg \cdot dL^{-1}$
Female	For ≤0.7	$144 \times (SCr/0.7)^{-0.329} \times 0.993^{Age}$
Female	For >0.7	$144 \times (SCr/0.7)^{-1.209} \times 0.993^{Age}$
Male	For ≤0.9	$141 \times (SCr/0.9)^{-0.411} \times 0.993^{Age}$
Male	For >0.9	$141 \times (SCr/0.9)^{-1.209} \times 0.993^{Age}$
		Serum Cystatin (SCys), $mg \cdot dL^{-1}$
Female or Male	For ≤0.8	$133 \times (SCys/0.8)^{-0.499} \times 0.996^{Age} \times 0.932$ if female
Female or Male	For >0.8	$133 \times (SCys/0.8)^{-1.328} \times 0.996^{Age} \times 0.932$ if female

Data from Inker et al. (2012).

Table S2. Effect of exercise tests on estimated glomerular filtration rate.

	Maximal		80% of maximal heart rate		60% of maximal heart rate	
	Estimated by Cystatin C, mL·min·1.73 m^2					
ID	Basal	Final	Basal	Final	Basal	Final
1	105.2	118.7	111.2	117.0	116.4	115.6
2	71.8	72.9	78.9	89.3	70.9	116.1
3	76.7	81.1	76.7	91.3	83.5	82.3
4	112.3	112.9	104.8	101.5	104.1	109.1
5	120.5	147.4	48.4	105.9	103.5	110.3
6	106.6	83.0	101.3	105.7	99.3	82.0
7	107.8	111.8	93.1	71.4	109.4	91.2
8	91.7	106.6	97.6	129.1	105.2	112.7
9	70.3	64.7	102.1	105.0	75.0	108.7
10	106.3	95.6	77.9	95.1	93.0	89.9
11	74.6	100.3	93.8	68.3	103.6	86.5
12	109.2	105.4	105.9	107.4	100.1	96.7
13	107.3	114.8	104.5	107.4	108.8	153.3
14	105.5	111.2	104.7	106.7	102.3	143.9
15	110.3	113.3	120.8	123.6	124.6	122.2
16	108.1	126.5	117.0	111.6	98.8	117.0
17	103.6	107.5	96.7	137.8	97.4	105.6
18	99.8	98.9	95.8	78.2	94.0	82.8
	Estimated by Creatinine, mL·min·1.73 m^2					
1	93.3	97.7	104.8	102.3	100.2	108.3

2	50.5	58.4	50.5	51.2	50.5	56.2
3	52.6	50.0	58.0	68.9	64.4	71.3
4	86.8	91.7	87.2	83.1	85.1	84.9
5	91.1	87.2	81.3	90.4	81.3	80.7
6	70.3	48.6	70.3	72.9	48.9	53.1
7	89.8	89.0	89.3	75.0	80.3	81.1
8	72.5	72.0	70.1	62.1	61.2	62.0
9	79.3	68.3	73.8	75.4	62.6	54.4
10	49.5	43.8	66.2	69.7	54.1	53.6
11	69.1	69.9	51.6	55.8	55.9	56.5
12	93.7	96.9	85.0	91.0	85.0	107.4
13	99.0	105.3	97.3	97.9	87.0	85.8
14	88.1	93.5	57.2	71.4	59.4	55.6
15	61.5	71.0	59.3	58.5	43.2	43.4
16	95.0	99.8	84.9	76.0	54.1	90.6
17	77.6	70.7	96.5	99.0	71.1	67.8
18	57.6	52.2	51.3	68.1	65.4	48.3
